# Physicians’ Lease Committee, Chicago Rotary Club

**Published:** 1917-08

**Authors:** R. R. Denny

**Affiliations:** Chairman; Chicago Rotary Club; Care Dennos Food Sales Co., Chicago, Ill.


### Journal-Record of Medicine

Successor to Atlanta Medical and Surgical Journal, Established 1855
and Southern Medical Record, Established 1870

**OWNED BY THE ATLANTA MEDICAL JOURNAL COMPANY**

PUBLISHED MONTHLY

*Official Organ Fulton County Medical Society, State Examing Board, Atlanta, Birmingham and Atlantic Railroad Surgeon's Association, Chattahoochee Valley Medical and Surgical Association, Etc.*

A. W. STIRLING, M. D., C. M., D. P. H.; J. S. HURT, B. Ph., M.D.
GEO. M. NILES, M. D., W. J. LOVE, M. D., (Ala.); Associate Editors

E. W. ALLEN, Business Manager

**COLLABORATORS**

W. F. WESTMORELAND, M. D., General Surgery
F. W. McRAE, M. D., Abdominal Surgery
ALLEN H. BUNCE, Medical Societies
E. B. BLOCK, M. D., Diseases of the Nervous System
MICHAEL HOKE, M. D., Orthopedic Surgery
CYRUS W. STRICKLER, M. D., Legal Medicine and Medical Legislation
E. C. DAVIS, A. B., M. D., Obstetrics
E. G. JONES, A. B., M. D., Gynecology
ALLEN H. BUNCE, M. D., Pathology and Bacteriology
R. T. DORSEY, JR., B. S., M. D., Medicine
L. M. GAINES, A. B., M. D., Internal Medicine
GEO. C. MIZELL, M. D., Diseases of the Stomach and Intestines
L. B. CLARKE, M. D., Pediatrics
EDGAR PAULLIN, M. D., Opsonic Medicine
THEODORE TOEPEL, M. D., Mechano Therapy
E. G. BALLENGER, M. D., Diseases of the Genito-Urinary Organs

VOL. LXIV. ATLANTA, GA., AUGUST, 1917. NO. 5

### PHYSICIANS' LEASE COMMITTEE CHICAGO ROTARY CLUB.

Aug. 24, 1917.

Dear Editor:

Enclosed you will find copy of a letter which is self-explanatory.

It occurred to us that you might co-operate also in helping this Committee get definite information on the points in question.

If you can give this matter proper publicity, asking your subscribers to address their answer to R. R. Denny, Chairman of the Committee, care the Dennos Food Sales Company, Chicago, it will be deeply appreciated.

We trust that the proposition is of sufficient importance to command the attention of your editor and space in your publication.

Any recognition that you give this matter would be gladly noted by the Committee.

Very truly yours

R. R. Denny, Chairman.

Aug. 18, 1917

Dear Doctor:

The Chicago Rotary Club has learned that a great number of physicians, who have enlisted for service during the present war, are embarrassed by unexpired leases. In certain cases, such corporations from whom they rent have refused to cancel leases. It seems to the Chicago Rotary Club that when physicians are so much needed in the United States Army, every effort should be made to relieve them of contracts rightfully binding in times of peace, but which might better be waived in times of national peril.

We all know that the physician giving up an established practice to enlist makes perhaps the biggest sacrifice of us all, because his business depends absolutely on personal contact. The day he leaves, his business ceases. But his lease goes on. Yet our country is calling for more physicians, and many patriotic doctors everywhere are trying to arrange their affairs to go.

It is possible to create a strong public opinion favoring the canceling of leases in such cases. If advisable, the matter can be carried for consideration to Congress. But first, the Physicians' Lease Committee wants figures and facts. We are sending this letter to 20,000 physicians scattered all over the United States. May we ask you personally to help us by promptly filling out and mailing back to us the enclosed postal card? Kindly do it today.

Your prompt co-operation will place in the hands of your committee the necessary data for an effective presentation of the facts before proper legislative bodies.

We want to help. We believe, in fairness to all, a great work can be done. We know that you will be glad to mail the card today. When we receive it, you will have our earnest thanks for your co-operation.

Yours very sincerely,

CHICAGO ROTARY CLUB,

R. R. Denny, Chairman.

Care Dennos Food Sales Co., Chicago, Ill.

